# Suppressor of Cytokine Signaling 3 (SOCS3) Degrades p65 and Regulate HIV-1 Replication

**DOI:** 10.3389/fmicb.2019.00114

**Published:** 2019-01-31

**Authors:** Vikas Sood, Sneh Lata, Vishnampettai G. Ramachandran, Akhil C. Banerjea

**Affiliations:** ^1^Department of Microbiology, University College of Medical Sciences and GTB Hospital, New Delhi, India; ^2^Virology Lab II, National Institute of Immunology, New Delhi, India

**Keywords:** HIV-1, SOCS3, p65, ubiquitination, NF-κB

## Abstract

Human Immunodeficiency Virus-1 (HIV-1) is known to induce the expression of SOCS3 which is a negative feed-back regulator of inflammatory responses. Here, we demonstrate that reactivation of latent HIV-1 leads to degradation of SOCS3 at early time points. Interestingly, SOCS3 degradation following transfection of HIV-1 RNA as well as polyIC in THP-1 cells further confirmed the role of viral RNA signaling in SOCS3 biology. Degradation of SOCS3 contributes toward viral RNA induced inflammatory responses. NF-κB signaling is also induced upon HIV-1 infection which leads to the production of pro-inflammatory cytokines to control the viral spread. Further investigations revealed that SOCS3 inhibits the expression and activity of p65 by interacting with it and inducing its ubiquitin-dependent proteasomal degradation. SH2 domain was critical for SOCS3-p65 interaction and p65 degradation. We also found that expression of SOCS3 promotes HIV-1 replication. Thus, HIV-1 downregulates SOCS3 in early phase of infection to promote inflammatory responses for large production of activated cells which are suitable for viral spread and induces SOCS3 later on to limit inflammatory responses and ensure viral survival.

## Introduction

Cytokines are secreted from the cells in response to diverse range of stimuli including microbial infections and inflammation. They bind to their respective receptors leading to the induction of downstream signaling cascade. Though induction of cytokines is crucial against microbial defense, termination of cytokine signaling is equally critical as activation of uncontrolled pathways leads to excessive inflammation which is detrimental to the host (Murray and Smale, 2012). Therefore, tight regulation of signaling pathways is important for maintaining cellular homeostasis. Among several pathways, nuclear factor kappa B (NF-κB) activation leads to the induction of cytokines and inflammatory responses. This pathway is evolutionary conserved and is essential for development and maintenance of immune-homeostasis (Hayden and Ghosh, 2011). This is tightly regulated by IkB kinase (IKK) complexes. In resting cells, IkBα interacts with p65 and sequesters it in the cytoplasm. However, during activation, IKK complex phosphorylates and ubiquitinates IkBα leading to its degradation and translocation of p65–p50 heterodimers to the nucleus which induces the transcription of their target genes. Interestingly, IkBα is one of the transcriptional targets of p65 which is rapidly synthesized upon NF-κB activation. Upon resynthesis, IkBα migrates to the nucleus where it binds to p65 and shuttles it back to the cytoplasm (Hayden and Ghosh, 2008). Apart from IkBα, NF-κB signaling is also regulated by multiple diverse pathways like akirin family of proteins, MAPK-activated protein kinase-2 (MK2) and p38 (Sun et al., 1993; Saccani et al., 2002; Goto et al., 2008). It has been also shown that several proteins like ODLIM2 and SOCS1 can induce p65 ubiquitination and proteasomal degradation leading to termination of NF-κB signaling (Ryo et al., 2003; Saccani et al., 2004; Tanaka et al., 2007; Strebovsky et al., 2011).

The SOCS family proteins are intracellular proteins which can regulate the cellular response to cytokines. The family consists of eight members (SOCS1-7 and CIS) having a central SH2 domain and a carboxy-terminal SOCS-box. Expression of most of the SOCS proteins is induced by cytokines and they act as negative feed-back regulator of JAK-STAT cytokine signaling pathway. Both SOCS1 and SOCS3 have been found to interact with JAK2 and terminate its activity (Endo et al., 1997). SOCS1 and SOCS3 also possess a kinase inhibitory region (KIR) which acts as pseudo-substrate for JAKs thereby terminating the signaling by a separate mechanism. JAK-STAT pathway is also one of the most important pathways which leads to the induction of cellular antiviral response. Viral infection leads to the production of type 1 interferons (IFNs) which activate JAK-STAT pathway resulting in the formation of ISGF3 complex which induces interferon stimulated genes (ISGs) Thus, SOCS family of proteins could modulate antiviral response via JAK-STAT pathway.

The possible role of SOCS3 in regulating cellular antiviral pathways was realized after a decade of discovery of SOCS proteins (Pauli et al., 2008). Recently, it was shown that influenza virus could inhibit IFN-β1 signaling via induction of SOCS3. Likewise, various other viruses ranging from Hepatitis to HIV-1 were also shown to induce the expression of SOCS family proteins, especially SOCS3 (Akhtar and Benveniste, 2011). Also, various proteins of SOCS family like SOCS1, SOCS2, and SOCS3 are known to regulate HIV-1 pathogenesis at various steps of viral replication suggesting critical role of SOCS proteins in HIV-1 pathogenesis (Ryo et al., 2008; Cheng et al., 2009; Akhtar et al., 2010). Furthermore, viral proteins like HIV-1 Tat has been reported to induce the expression of SOCS2 and SOCS3 which impair IFN-γ and IFN-β1 signaling, respectively (Cheng et al., 2009; Akhtar et al., 2010). In contrast, SOSC1 and SOCS3 protein levels have been found to be lower in CD4+ T-cells of HIV infected patients than in healthy controls (Miller et al., 2011). There is no clear explanation for these contrasting observations till now. So, we planned to investigate the expression levels of SOCS3 protein at different time points of viral replication. We found that SOCS3 protein expression is downregulated in early phase of HIV-1 life cycle followed by an increase in SOCS3 levels at later stages of viral replication. Viral RNA was found to be responsible for decreasing the expression of SOCS3. Then we wanted to figure out the significance of HIV-1 mediated differential regulation of SOCS3 expression at different stages of viral life cycle. Since HIV-1 Tat is known to induce NF-κB signaling (Dandekar et al., 2004; Fiume et al., 2012) and also Tat induces SOCS3 in NF-κB dependent manner (Akhtar et al., 2010), we investigated the effect of SOCS3 on NF-κB signaling. We observed that SOCS3 causes degradation of p65 via ubiquitin-mediated proteasomal pathway, thereby inhibiting NF-κB signaling. Hence, our study explains the differential regulation of SOCS3 during HIV-1 infection and uncovers a novel role of SOCS3 in the regulation of NF-κB signaling and host immune evasion of HIV-1.

## Materials and Methods

### Cell Lines

HEK-293T cells, CHME3 cells and Murine Embryonic Fibroblasts (MEFs) were grown in Dulbecco’s modified Eagle’s medium (DMEM; Invitrogen, Life Technologies) while U1 (monocytic cell line latently infected with HIV-1), THP-1, and U937 cells were grown in RPMI (Roswell Park Memorial Institute) 1640 supplemented with 10% fetal bovine serum (FBS; HyClone) and 100 units penicillin, 0.1 mg streptomycin and 0.25 μg amphotericin B per ml at 37°C in the presence of 5% CO_2_ in a humified incubator.

### Antibodies and Plasmids

Antibodies against SOCS3, p65 and GAPDH were obtained from Cell Signaling Technology. Anti-p65 for immunoprecipitation and anti-SOCS1 antibody was purchased from Santa Cruz Biotechnology. Anti-Myc and anti-HA antibodies were obtained from Clontech. Anti-flag antibody was obtained from Sigma. HIV-1 p24 antibody was obtained from NIH AIDS reagents program, United States. HRP-coupled secondary antibodies were obtained from Jackson ImmunoResearch. pCMV-Myc plasmid was obtained from Clontech and SOCS3, SOCS3 mutants and SOCS7 genes were cloned into this plasmid. Sequence of all the primers used for cloning is provided in Supplementary Table S1. Renilla luciferase plasmid was a kind gift from Vivek Natrajan, IGIB, Delhi, India. Flag-Ikkα and Flag-Ikkβ were kindly gifted by Dr. Thomas D. Gilmore, Boston University, Biology Department, Boston. NF-κB-Luc was obtained from Stratagene. SOCS3-luc reporter plasmid was a kind gift from Dr. Kakoli Ghoshal, National Institute of Immunology, New Delhi, India. Sources of other plasmids used in the experiments are listed in Supplementary Table S2.

### Cell Treatment, Virus Infection, and Plasmid Transfection

For viral infections, cells were either seeded in six or twelve well plate 1 day before infection. Viral soup was added to cells in the presence of 6 μg/ml polybrene for 4 h at 37°C. Following infection, cells were washed twice and then incubated in humidified CO_2_ incubator for indicated time points. Lipofectamine 2000 (Invitrogen) was used for plasmid transfections in HEK-293T and CHME3 cells. For virus production, plasmids were transfected in 100 mm HEK-293T cells using calcium chloride method. TNFα treatment was given to U1 cells for the different time periods to induce the viral production.

### Western Blotting

Cells transfected with various plasmids or infected with different viruses were washed in PBS and lysed in RIPA lysis buffer. Protein was quantified using the BCA protein assay kit (Pierce, Thermo Scientific, United States). Cell lysates were processed by SDS-PAGE followed by western blotting as described previously (Lata et al., 2015).

### Whole Cellular RNA Isolation and Transfection

Whole cellular RNA was isolated from TNFα induced U1 cells and U937 cells as described earlier (Lata et al., 2015) and used to stimulate THP-1 cells (Pauli et al., 2008). The U1 cells were treated with TNFα for 24 h, and total RNA was isolated using Trizol reagent as described by the manufacturer (Invitrogen). RNA was transfected into THP-1 cells using Lipofectamine 2000 (Invitrogen) using manufacturer protocol.

### Dual Luciferase Reporter Assay

Luciferase assays were performed in HEK-293T cells using commercially available dual luciferase reporter assay kit (Promega). During transfections an internal control plasmid encoding renilla luciferase (pTKrl) was used. An empty pcDNA3.1 vector was added to normalize the amount of DNA transfected in each well. Twenty-four hours post transfection, cells were lysed in reporter lysis buffer and analyzed using multimode reader (Tecan) as described previously (Lata et al., 2015).

### Co-immunoprecipitation

The protein–protein interaction was studied by co-immunoprecipitation using Pierce^TM^ Direct IP kit (Thermo Scientific). HEK-293T cells were transfected with desired plasmids. After 24 h of transfection, cells were harvested and washed with PBS. Cells were lysed in cell lysis buffer and antibody conjugated agarose resin was added. It was rotated overnight at 4°C. After incubation, resin was pelleted and washed with wash buffer. The immunoprecipitated proteins were eluted using elution buffer. The aqueous solution containing eluted proteins was boiled with SDS–PAGE loading buffer for 5 min and analyzed by western blotting.

### *In vitro* Ubiquitination Assay

HEK-293T cells were co- transfected with p65, SOCS3, and His-Ub (6X histidine-ubiquitin) plasmid. Twenty four hours post transfection cells were incubated with MG132 for further 8 h. After incubation, Ubiquitination assay was performed as described earlier (Lata et al., 2015).

### VSV-G-Pseudotyped pNL4-3 Virus Preparation

To prepare the virus, 18 mg of pNL4-3 and 2 mg of VSV-G-expressing plasmid were transfected in a 100-mm cell culture dish of HEK-293T cells using Lipofectamine 2000 (Invitrogen). Medium was replaced with fresh complete DMEM after 6 h of transfection. The supernatant containing viral particles was collected after 48 h. The collected virus supernatant was filtered through a 0.45-mm-pore-size filter, and an aliquot was used for p24 assays using β-galactosidase staining of HIV-1 reporter cell line TZM-bl. The viral stock was stored at -80°C.

### Statistical Analysis

Data obtained were represented as mean ± SEM. *P*-value was calculated by a two-tailed student *t*-test. Only values of *P* < 0.05 were considered significant.

## Results

### Reactivation of HIV-1 in Latently Infected Monocytes Leads to Rapid Degradation of SOCS3

**FIGURE 1:**
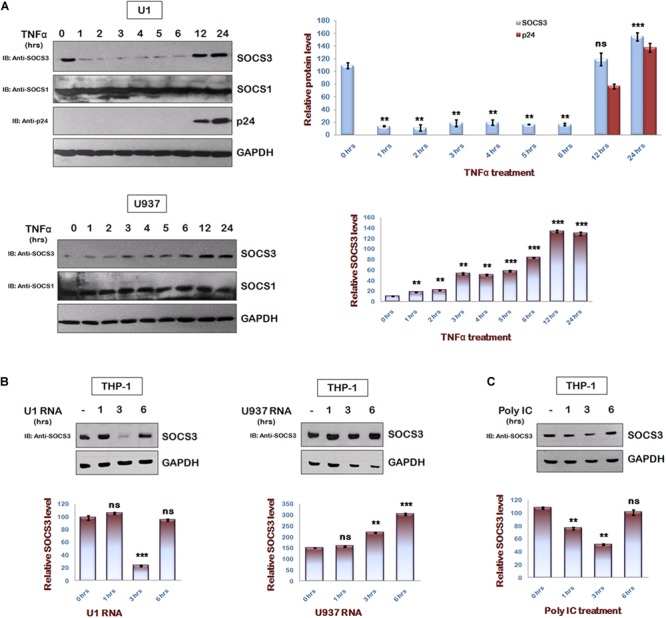
HIV-1 regulates SOCS3 expression which is mediated by Viral RNA in early phase of replication. **(A)** U1 and U937 cells were treated with TNFα (20 ng/ml) for different time periods as indicated. Cells were harvested and lysed in RIPA lysis buffer. Cell lysates were analyzed by western blotting for SOCS3, SOCS1, and p24 using their respective antibodies. **(B)** HIV RNA as a part of total RNA (30 μg/ml) isolated from TNFα (20 ng/ml) induced U1 cells and U937 total RNA (30 μg/ml; control) were transfected into THP-1 cells and lysates were prepared at different time points as shown. Cell lysates were subjected to western blot analysis for SOCS3 using anti-SOCS3 antibody. **(C)** THP-1 cells were transfected with PolyIC (30 μg/ml) and lysates were prepared at different time points as indicated. Lysates were analyzed for SOCS3 using anti-SOCS3 antibody. GAPDH was probed as loading control using anti-GAPDH antibody. Densitometric analysis of SOCS3 and p24 levels was done by ImageJ software and values are represented as bar diagram. The values represent the mean + SEM of three independent experiments. *P*-value was calculated by student *T*-test (^∗^*p* < 0.05, ^∗∗^*p* < 0.01, ^∗∗∗^*p* < 0.001).

To understand the regulation of SOCS3 expression during HIV-1 replication, we studied endogenous levels of SOCS3 in U1 cells after TNFα treatment (Duh et al., 1989; Griffin et al., 1989). It was observed that TNFα induced HIV-1 reactivation in U1 cells led to rapid degradation of SOCS3 upto 6 h of TNFα treatment followed by an increase in expression of SOCS3 at later time points (Figure 1A upper panel). This effect was specific to SOCS3 as we could not detect any change in levels of SOCS1. TNFα treatment of control U937 cells led to induction of SOCS3 (Figure 1A lower panel) thereby suggesting that early events in reactivation of HIV-1 leads to the specific degradation of SOCS3. Expression of SOCS3 is already known to be induced by HIV-1 Tat (Akhtar et al., 2010). To further find out the viral factor responsible for downregulation of SOCS3 at early time points, we isolated total RNA from TNFα induced U1 cells containing HIV-1 RNA and U937 cells and transfected into THP-1 cells. As expected, we observed rapid degradation of SOCS3 in response to HIV-1 RNA as compared to RNA from uninfected cells suggesting that viral RNA induces the specific degradation of SOCS3 (Figure 1B). To further validate our findings, we transfected THP-1 cells with polyIC (viral RNA mimic). polyIC was also found to induce the degradation of SOCS3 (Figure 1C). PolyIC mediated degradation was also observed in HeLa cells and Mouse peritoneal macrophages (Supplementary Figure S1). All these results confirmed our findings that signaling pathways induced by viral RNA leads to the rapid degradation of SOCS3.

### SOCS3 Is a Negative Regulator of NF-κB Signaling

**FIGURE 2:**
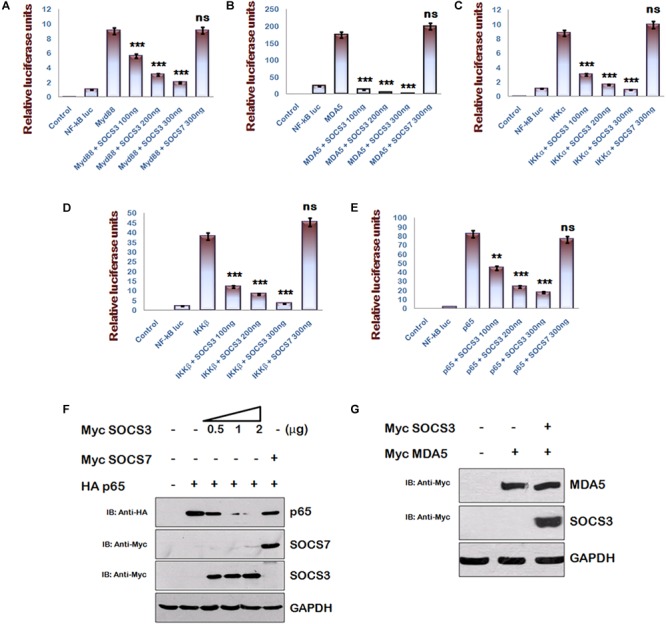
SOCS3 inhibits NF-κB pathway induced by Myd88, MDA5, IKKα, IKKβ, and p65 via degradation of p65. **(A–E)** HEK293T cells were co-transfected with an NF-κB-luc reporter plasmid (100 ng), renilla luciferase, Flag-MyD88, Flag-IKKα, Myc-MDA5, Flag-IKKβ, pCMV-p65 together with an empty vector or SOCS3 or SOCS7 encoding plasmid as shown and analyzed for NF-κB-dependent luciferase activity (fold induction) by dual luciferase reporter assay. The values represent the mean ± SEM of three independent experiments. *P*-value was calculated by student *T*-test (^∗^*p* < 0.05, ^∗∗^*p* < 0.01, ^∗∗∗^*p* < 0.001). **(F)** HA-p65 was co-transfected with Myc-SOCS3 or Myc-SOCS7 in HEK-293T cells and analyzed by western blotting for the levels of p65using anti-HA antibody. **(G)** Myc-MDA5 was co-transfected with Myc-SOCS3 in HEK-293T cells and analyzed for the levels of MDA5 in presence and absence of SOCS3 using anti-Myc antibody by western blotting. GAPDH was probed as loading control using anti-GAPDH antibody.

Multiple signaling pathways like NF-κB and AP-1 converge to drive IFN-β1 expression, but inhibition of IFN-β1 signaling via SOCS3 was studied in context of JAK-STAT signaling pathway (Akhtar et al., 2010) only. Since NF-κB signaling is a central pathway that drives the inflammatory responses, we studied the components of NF-κB signaling pathway and utilized NF-κB luciferase reporter plasmid to investigate the significance of HIV-1 mediated downregulation of SOCS3 expression at early stage of its life cycle using another gene of SOCS family, i.e., SOCS7 as negative control. We found that SOCS3 can inhibit MyD88-mediated NF-κB activation in a dose-dependent manner while SOCS7 cannot do so (Figure 2A). Since Myd88 regulates multiple signaling pathways during TLR induction, we sought to characterize SOCS3-mediated NF-κB inhibition parallel and downstream of MyD88 also. So, we employed other inducers of NF-κB signaling like MDA5, IKKα, and IKKβ to activate NF-κB reporter. We observed that SOCS3 inhibits NF-κB reporter activity with all the above mentioned inducers while SOCS7 is not able to do so (Figure 2B–D). This indicates that either SOCS3 inhibits IKK complex or inhibitory effect of SOCS3 lies further downstream. Then we studied pathways downstream of IKK and observed that SOCS3 could inhibit p65-mediated NF-κB activation (Figure 2E) which suggests that SOCS3 might act at the level of p65 (important subunit of NF-κB complex) to inhibit NF-κB signaling. In contrast, co- transfection of SOCS7 did not result in inhibition of p65 mediated NF-κB activation indicating that SOCS3 specifically regulates the activity of p65 (Figure 2E).

### SOCS3 Interacts With/Degrades p65 via Its SH2 Domain

Since SOCS family proteins are known to degrade their targets (Piessevaux et al., 2008) and SOCS1 is already reported to degrade p65 (Strebovsky et al., 2011), we co-transfected constant amounts of HA-p65 and increasing amounts of Myc-SOCS3 in HEK-293T cells and estimated the expression levels of p65. We observed that exogenous expression of SOCS3 led to rapid degradation of p65 in a dose-dependent manner (Figure 2F). As discussed earlier, we also confirmed that exogenous expression of SOCS7 did not affect the levels of p65 confirming the specificity of SOCS3 in regulating the levels of p65. To rule out any SOCS3 mediated non-specific degradation in over-expression system, we co-transfected Myc-MDA5 [one of the important members of RIG like receptor (RLR) pathway] with Myc-SOCS3 in HEK-293T cells. We observed that unlike p65, levels of MDA5 were not affected by SOCS3 (Figure 2G) further suggesting that SOCS3 regulates p65 in a highly specific manner.

**FIGURE 3:**
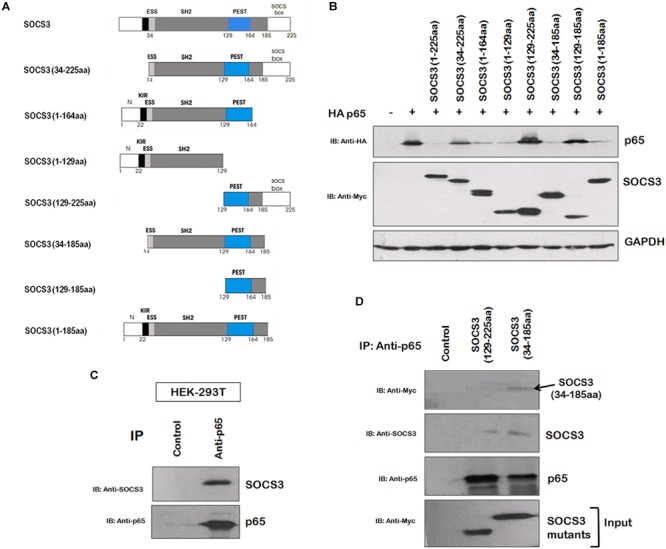
SOCS3 causes degradation of p65 by interaction through its SH2 domain. **(A)** Several truncated mutants of SOCS3 were cloned in pCMV-myc vector to identify domain necessary for inducing p65 degradation. **(B)** Myc-SOCS3 or its mutants were co-transfected along with HA-p65 in HEK-293T cells and analyzed for the expression of p65 using anti-HA antibody by western blotting. SOCS3 and SOCS7 were analyzed by anti-Myc antibody. GAPDH was probed as loading control using anti-GAPDH antibody. **(C)** HEK-293T cell lysate was incubated with anti-p65 conjugated agarose resin overnight at 4°C. The proteins bound to the resin were eluted and analyzed by western blotting for SOCS3 and p65 using their respective antibodies. **(D)** HEK-293T cells were transfected with Myc-SOCS3 (129–225 aa) mutant lacking SH2 domain and Myc-SOCS3 (34–185 aa) mutant (positive control) and lysates were prepared followed by incubation with anti-p65 conjugated agarose resin overnight at 4°C. The proteins bound to the resin were eluted and analyzed by western blotting for SOCS3, Myc-SOCS3 (129–225 aa), Myc-SOCS3 (34–185 aa) and p65 using their respective antibodies.

In order to gain insights into the domains of SOCS3 responsible for p65 degradation, we cloned several truncated mutants of SOCS3 lacking one or more domains with myc tag and studied their effect on p65 stability (Figure 3A). The mutants, lacking SH2 domain, were found to be unable to degrade p65 (Figure 3B) suggesting that SH2 domain of SOCS3 plays a vital role in degradation of p65. As SH2 domain of SOCS3 mediates its interaction with its target proteins (Nicholson et al., 2000), we investigated the interaction between SOCS3 and p65. HEK 293T cells were lysed and lysates were incubated with anti-p65 bound agarose resin overnight at 4°C and the immunoprecipitated proteins bound to the resin were eluted and analyzed by western blotting. SOCS3 was found to be interacting with p65 (Figure 3C). Also, SOCS3 mutant lacking SH2 domain was not able to interact with p65 (Figure 3D).

### SOCS3 Induces Ubiquitination of p65

**FIGURE 4:**
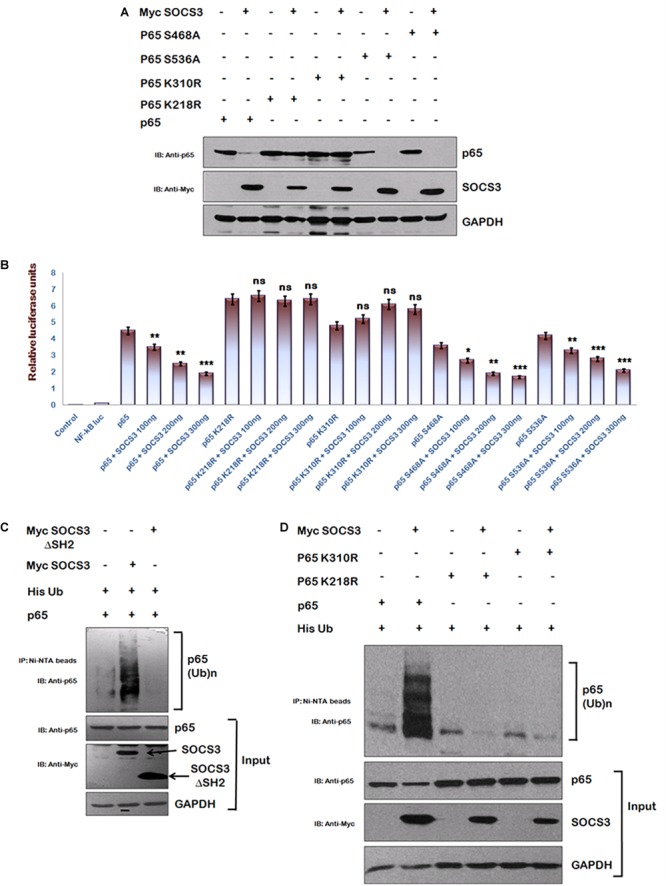
SOCS3 causes ubiquitination of p65. **(A)** HEK-293T cells were transfected with p65, p65 K218R, p65 K310R, p65 S468A, and p65 S536A encoding plasmids in presence or absence of Myc-SOCS3 for 24 h. Cells were lysed and subjected to western blotting using anti-p65 antibody. **(B)** HEK-293T cells were transfected with NF-κB-luc reporter plasmid, renilla luciferase, p65, p65 K218R, p65 K310R, p65 S468A, and p65 S536A encoding plasmids along with different doses of Myc-SOCS3 as shown. After 24 h of transfection, luciferase activity was assayed using dual luciferase assay reagents by luminometer. The values represent the mean ± SEM of three independent experiments. *P*-value was calculated by student *T*-test (^∗^*p* < 0.05, ^∗∗^*p* < 0.01, ^∗∗∗^*p* < 0.001). **(C)** HA- p65 and His-Ubiquitin were co-transfected in HEK-293T cells in the presence or absence of Myc-SOCS3 and Myc-SOCS3ΔSH2 (negative control). The cells were incubated with MG132 for 8 h and cell lysates were prepared. Immune complexes were pulled down using Ni-NTA beads and analyzed by western blotting using anti-p65 antibody. **(D)** HEK-293T cells were transfected with His- ubiquitin, p65, p65 K218R, and p65 K310R encoding plasmids along with Myc-SOCS3. The cells were incubated with MG132 for 8 h and cell lysates were analyzed as explained above in **(C)**. SOCS3 was probed with anti-Myc antibody and GAPDH was blotted as loading control using anti-GAPDH antibody.

To identify regions of p65 required for SOCS3 mediated degradation, we utilized several phosphorylation and acetylation defective mutants of p65. We observed that whereas SOCS3 caused a rapid degradation of phosphorylation mutants of p65 (S468A and S536A) similar to wild type p65, acetylation mutants of p65 (K218R and K310R) were refractory to SOCS3 mediated degradation (Figure 4A). Similar experiment was performed on the NF-κB reporter plasmid. Transactivation of NF-κB reporter by p65 or phosphorylation defective p65 mutants was suppressed by SOCS3 in dose-dependent manner while SOCS3 could not affect transactivation of NF-κB reporter by acetylation defective mutants of p65 (Figure 4B) suggesting that these lysine residues might be responsible for SOCS3 mediated p65 degradation.

Since SOCS family of proteins induces the ubiquitination of their target proteins (Piessevaux et al., 2008) and lysine residues are the potential sites for both acetylation and ubiquitination of p65 (Li et al., 2012), we investigated the ubiquitination status of p65 in the presence of SOCS3. His-Ub was co-transfected along with either wt Myc-SOCS3 or one of the SOCS3 mutants lacking SH2 domain (SOCS3 129–225 aa) along with p65 in HEK-293T cells and cells were treated with MG132. Following the incubation, immune complexes were pulled down using Ni-NTA beads and analyzed by immunoblotting for ubiquitinated p65. As expected, expression of SOCS3 led to enhanced ubiquitination of p65 whereas SOCS3 mutant lacking SH2 domain was unable to do so (Figure 4C). In order to further verify our results, we studied the effect of SOCS3 on ubiquitination levels of acetylation defective mutants of p65. In agreement with our earlier data, we could not observe any ubiquitination of acetylation defective mutants of p65 (Figure 4D). This indicates that the lysine residues K218 and K310 of p65 are ubiquitinated in the presence of SOCS3.

### SOCS3 Is Required for HIV-1 Pathogenesis

**FIGURE 5:**
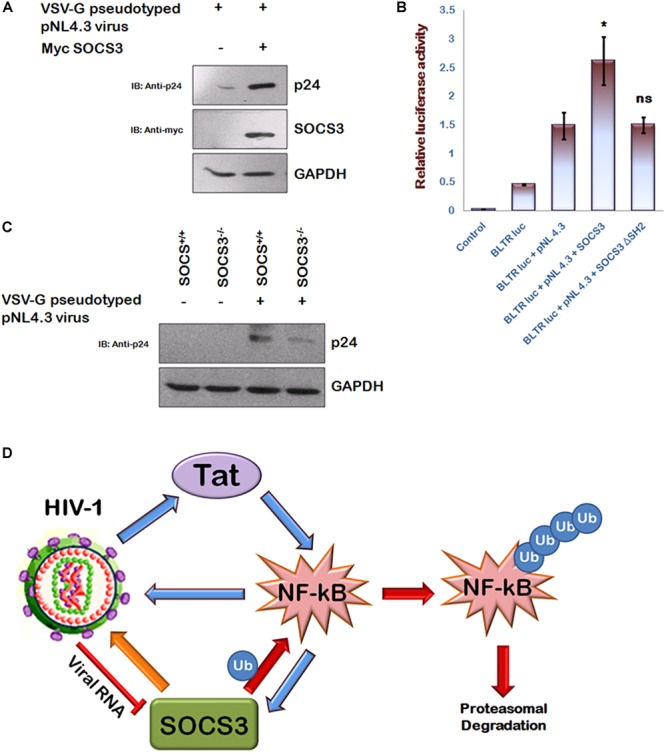
SOCS3 promotes viral replication. **(A)** CHME3 cells were infected with VSV-G pseudotyped pNL4-3 virus in presence or absence of Myc-SOCS3. After 24 h of infection, cells were lysed and subjected to western blotting using anti-p24 and anti-SOCS3 antibodies. **(B)** HEK-293T cells were transfected with B LTR-luc reporter plasmid (250 ng), renilla luciferase, pNL4.3, Myc-SOCS3, and Myc-SOCS3 ΔSH2 as shown for 24 h. Empty vector was used to equalize the amount of DNA transfected. After 24 h of transfection, luciferase activity was assayed using dual luciferase assay reagents by luminometer. The values represent the mean + SEM of three independent experiments. P-value was calculated by student T-test (^∗^p < 0.05, ^∗∗^p < 0.01, ^∗∗∗^p < 0.001). **(C)** SOCS3+/+ and SOCS3–/– MEFs were infected with VSV-G pseudotyped pNL4.3 virus for 24 h and lysed. Cell lysates were analyzed for p24 using anti-p24 antibody. GAPDH was probed as loading control using anti-GAPDH antibody. **(D)** Mechanistic model of inter-regulation of HIV-1, SOCS3, and NF-κB.

In agreement with the previous reports (Akhtar et al., 2010; Fiume et al., 2012), we also observed that HIV-1 Tat enhances p65 induced activation of NF-κB promoter (Supplementary Figure S2) and activates SOCS3 promoter (Supplementary Figure S3) thereby establishing the critical role of Tat in inducing the expression of SOCS3. Since NF-κB is also well known to increase HIV-1 LTR transactivation resulting in enhanced viral replication (Dandekar et al., 2004) and we earlier observed that SOCS3 induces degradation of p65, an important subunit of NF-κB (Figure 2F), so we sought to study the effect of SOCS3 on HIV-1 replication. For HIV-1 infection based studies, we utilized human microglial cell line (CHME3) which are the natural targets of HIV-1 infections (Henning et al., 2010) recently described by us (Lata et al., 2015). Expression of SOCS3 followed by VSV-G pseudotyped HIV-1 infection in CHME3 cells led to enhanced replication of HIV-1 as observed by increased levels of p24 (Figure 5A). This was further confirmed by using HIV-1 LTR luciferase reporter plasmid and SOCS3 ΔSH2 mutant as negative control. SOCS3 was found to enhance the LTR luciferase activity induced by the virus while SOCS3 ΔSH2 mutant was unable to do so (Figure 5B). To further validate our findings, SOCS3^-/-^ knockout MEFs (Mouse Embryonic Fibroblasts) and wild type SOCS3^+/+^ MEFs were used. SOCS3^-/-^ MEFs are reported to have higher expression of p65 than in SOCS3^+/+^ MEFs (Lu et al., 2006). We also verified this observation (Supplementary Figure S4). These cells were infected with VSV-G pseudotyped HIV-1. The levels of p24 were found to be more in SOCS3^+/+^ MEFs than in SOCS3^-/-^ MEFs which indicates that the efficient HIV-1 replication requires SOCS3 (Figure 5C). Hence our data suggests that SOCS3 promotes HIV-1 replication which further induces the expression of SOCS3 creating a positive feed-back loop between HIV-1 and SOCS3 as shown in the proposed model (Figure 5D).

## Discussion

SOCS3 is the member of SOCS family of proteins which are functionally characterized to inhibit JAK-STAT signaling pathway, thereby suppressing the action of cytokines. SOCS3 has been shown to be induced by numerous viruses including Influenza, Hepatitis and HIV-1 (Akhtar et al., 2010). But HIV-1 patients have been found to have low levels of SOCS3 protein (Miller et al., 2011). To explain these contrasting observations, we studied the expression of SOCS3 in HIV-1 latently infected monocytic cells at different time points of TNFα mediated induction. For the first time, we report that expression of SOCS3 is differentially regulated by HIV-1 during its infection cycle. At the early stage, HIV-1 induces the downregulation of SOCS3 levels while the expression of SOCS3 is induced at the later stage, which is already known to be mediated by HIV-1 Tat (Akhtar et al., 2010). Since RNA of the virus is first released into the host cell after the fusion which is recognized by the specific cellular receptors resulting in the initiation of anti-viral immune response, we investigated the effect of viral RNA on the expression of SOCS3 to explain downregulation of SOCS3 in the initial phase of viral infection. A significant reduction in the levels of SOCS3 was observed in the presence of HIV-1 RNA.

To find out the significance of temporal regulation of SOCS3 expression by HIV-1, we first investigated the effect of SOCS3 on NF-κB signaling. Being a central pathway, NF-κB signaling is induced by a number of upstream pathways including innate immune signaling pathways which regulate inflammatory responses for controlling microbial invasion. However, like a double edged sword, these responses, if not controlled, can prove to be detrimental to the host itself. Regulation of inflammatory responses is a complex and highly regulated phenomenon and though much is known about the induction of these responses, how they are regulated temporally warrants further studies. In this report, we show that SOCS3 can inhibit NF-κB reporter gene activation in a dose-dependent manner by degrading p65 thereby downregulating NF-κB signaling. Our data also suggested that SH2 domain of SOCS3 is essential for binding with p65 and inducing its degradation. Mutational analysis of p65 to identify key residues that were critical for SOCS3 mediated degradation revealed that phosphorylation mutants (S468A and S536A) of p65 are degraded by SOCS3 with similar efficiency as wild type, while acetylation mutants of p65 (K218R and K310R) are not degraded suggesting a possible role of lysine residues in SOCS3 mediated p65 degradation probably by ubiquitination through proteasome. Further we show that wt SOCS3 but not SOCS3 lacking SH2 domain ubiquitinates p65 leading to its degradation via proteasomal pathway. Interestingly, one of the recent studies also supports our hypothesis indirectly where authors have studied the role of SOCS proteins in Fas induced cell death. In this study, authors have shown that levels of p65 are diminished in T- cells that stably express SOCS3 (Oh et al., 2012) suggesting the possible inhibitory role of SOCS3 on p65 stability.

The earlier reports showing the role of SOCS3 in viral pathogenesis point toward the modulation of JAK-STAT pathway only which is already a very well characterized phenomenon. Hence, we have uncovered a novel pathway of SOCS3 mediated inhibition of NF-κB signaling via ubiquitin-mediated degradation of p65. NF-κB is also well known to increase HIV-1 LTR transactivation due to the presence of two adjacent high affinity NF-κB binding sites in the enhancer region of LTR resulting in enhanced viral replication (Nabel and Baltimore, 1987). Conversely, HIV-1 is also reported to promote NF-κB signaling. Interestingly, it was observed that Jurkat cells stably expressing HIV-1 Tat shows constitutive activation of NF-κB (Scala et al., 1994). This finding was further validated and it has been shown that HIV-1 Tat binds to NF-κB enhancer sequences as well as associates with IkBα and p65 to activate NF-κB signaling (Dandekar et al., 2004; Fiume et al., 2012). So, to find out the possible advantage of HIV-1 mediated temporal regulation of SOCS3 to the virus itself, we investigated the effect of SOCS3 on HIV-1 infection. Here, we show that expression of SOCS3 leads to enhanced HIV-1 replication in HIV-1 permissive cell line (CHME3). Hence, we hypothesize that apart from inhibiting IFN-β1 signaling, termination of NF-κB signaling by SOCS3 might also play a vital role in SOCS3-mediated enhancement of viral replication by reducing host inflammatory responses.

Since HIV-1 Tat activates NF-κB signaling and induces the expression of SOCS3 in NF-κB dependent manner, it is very likely that the interplay between Tat-NF-κB-SOCS3 is also temporal in nature. HIV-1 Tat is one of the earliest proteins that is formed following viral infection and induces NF-κB signaling resulting in the inflammatory response and production of large number of activated cells which are favorable for viral replication. HIV-1 RNA also downregulates the expression of SOCS3 at early stage of infection to promote NF-κB signaling. Once optimal viral replication is achieved, more synthesis of HIV-1 Tat leads to the induction of NF-κB dependent genes including SOCS3 which then terminates IFN-β1 and NF-κB signaling thereby keeping the host anti-viral responses under check in order to facilitate the survival of the virus and further promote virus replication. Hence, we have identified a unique pathway where SOCS3 leads to ubiquitin- mediated degradation of p65 thereby acting as a negative feed-back inhibitor of NF-κB signaling that helps in viral immune evasion and promotes the replication of HIV-1 which induces NF-κB signaling and SOCS3 expression thereby further promoting viral replication (Figure 5D).

## Author Contributions

VS, SL, AB, and VR conceived the idea. VS and SL designed and performed the experiments. VS, SL, and AB wrote the manuscript.

## Conflict of Interest Statement

The authors declare that the research was conducted in the absence of any commercial or financial relationships that could be construed as a potential conflict of interest.
