# Supplementary material for: Suppressor of Cytokine Signaling 3 (SOCS3) Degrades p65 and Regulate HIV-1 Replication
Source: Front Microbiol. 2019 Jan 31;10:114. doi: 10.3389/fmicb.2019.00114 (PMC6365456; doi:10.3389/fmicb.2019.00114)
Supplement: Supplementary file 1 [file Data_Sheet_1.PDF]

Supplementary Table 1: Primers used for cloning SOCS3, deletion mutants of SOCS3 and SOCS7

Gene	Primers
**SOCS3**	**FP:** 5'-GGCGTCGACCATGGTCACCCACAGCAAGTTTC-3'
	**RP:** 5'-GGCGCGGCCGCTTAAAGCGGGGCATCGTACTG-3'
**SOCS3 (34-225)** (N-terminal and KIR domains deleted)	**FP:** 5'-GGCGTCGACCATGGTGGTGAACGCAGTGCGCAAGC-3'
	**RP:** 5'GGCGCGGCCGCTTAAAGCGGGGCATCGTACTG3'
**SOCS3 (1-164aa)** (Part of SH2 domain & full SOCS box deleted)	**FP:** 5'-GGCGTCGACCATGGTCACCCACAGCAAGTTTC-3'
	**RP:**5'-GGCGCGGCCGCTTAGGCTCTTCTGGGGGGACTCCCA-3'
**SOCS3 (1-129aa)** (PEST motif, part of SH2 domain & SOCS box deleted)	**FP:** 5'-GGCGTCGACCATGGTCACCCACAGCAAGTTTC-3'
	**RP:** 5'-GGCGCGGCCGCTTACGGCATGTAGTGGTGCACCAGCTT-3'
**SOCS3 (129-225aa)** (N-terminal, KIR, ESS & part of SH2 domain deleted)	**FP:** 5'-GGCGTCGACCATGCCCCCTGGAGCCCCCTCCTT-3'
	**RP:** 5'-GGCGCGGCCGCTTAAAGCGGGGCATCGTACTG-3'
**SOCS3 (34-184aa)** (N-terminal, KIR & SOCS box domain deleted)	**FP:** 5'-GGCGTCGACCATGGTGGTGAACGCAGTGCGCAAGC-3'
	**RP:** 5'-GGCGCGGCCGCTTAGGAGGAGAGGGGCCGGCTCA-3'
**SOCS3 (129-184aa)** (N-terminal, KIR, ESS, part of SH2 & SOCS box domains deleted)	**FP:** 5'-GGCGTCGACCATGCCCCCTGGAGCCCCCTCCTT-3'
	**RP:** 5'-GGCGCGGCCGCTTAGGAGGAGAGGGGCCGGCTCA-3'
**SOCS3 (1-185aa)** (SOCS box deleted)	**FP:** 5'-GGCGTCGACCATGGTCACCCACAGCAAGTTTC-3'
	**RP:** 5'-GCGAATTCTGATGGCAGGAAGAAGCGGAGAC3'
**SOCS7**	**FP:** 5'-GGCGTCGACCATGGTGTTCCGCAACGTGGGTC-3'
	**RP:** 5'-GGCGCGGCCGCCTACGTGGAGGGTTCCACCTCTTGC-3'

Supplementary Table 2: Sources of plasmids used in the experiments

S.N.	Plasmid Name	Investigator	Address of Investigator	Reference
1	pBlue3'LTR-luc-B.	Ben Berkhout	University of Amsterdam, Netherlands	1
**2**	pNL4-3	Malcolm Martin	NIAID, NIH, USA	2
**3**	pHEF-VSVG	Lung-Ji Chang	University of Florida, USA	3
**4**	6XHis-Ubiquitin	Dimitris Xirodimas	University of Dundee, UK	4
**5**	Myc-MDA5	Steve Goodbourn	St. George's, University of London, London, UK	5
**6**	HA-p65, HA-p65 S468A and HA-p65 S536A	Lienhard Schmitz	Justus Liebig University, Germany	6
**7**	pCMV-HA-Myd88 (Addgene 12287)	Bruce Beutler	The Scripps Research Institute, USA	7
**8**	pCMV4-p65 (Addgene 21966)<br>p65 (K218R) (Addgene 23247)<br>p65 (K310R) (Addgene 23250)	Warner Greene	Gladstone Institute of Virology and Immunology, University of California, USA	8<br>9<br>9

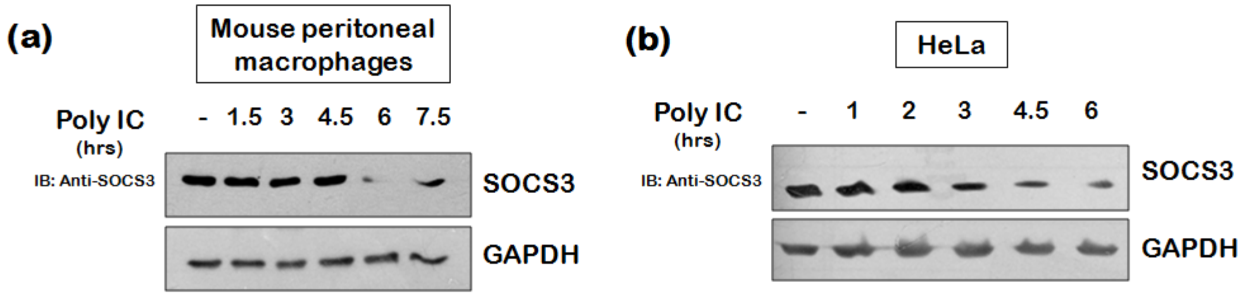

Supplementary Figure 1 (a,b) Mouse peritoneal macrophages and HeLa cells were transfected with PolyIC (30µg/ml) for different time periods as shown. Cell lysates were analyzed for SOCS3 using anti-SOCS3 antibody. GAPDH was probed as loading control using anti-GAPDH antibody.

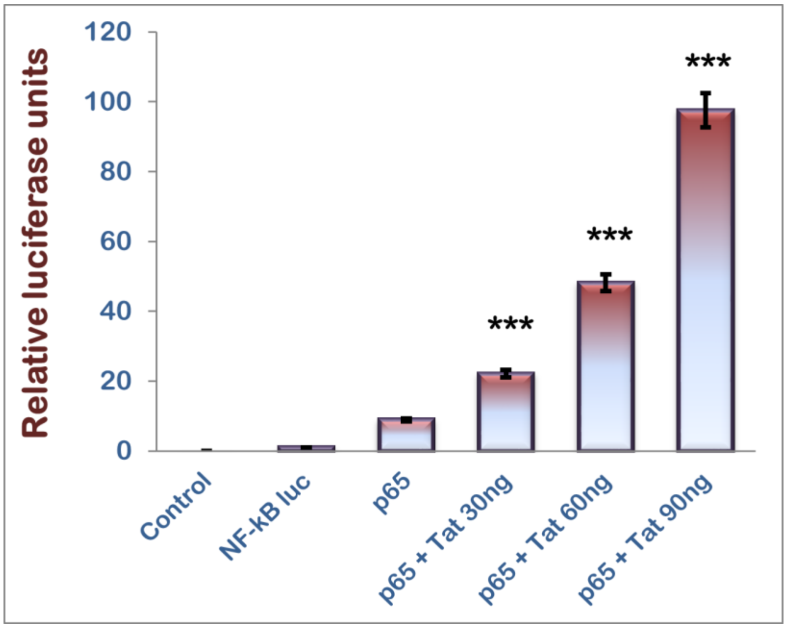

Supplementary Figure 2 HEK-293T cells were transfected with NF-kB-luc reporter plasmid (100ng), renilla luciferase, p65 along with different doses of Myc-Tat as shown for 24 hrs. Empty vector was used to equalize the amount of DNA transfected. After 24 hrs of transfection, luciferase activity was assayed using dual luciferase assay reagents by luminometer. The values represent the mean +SEM of three independent experiments. P value was calculated by student T test (*p<0.05, **p<0.01, ***p<0.001)

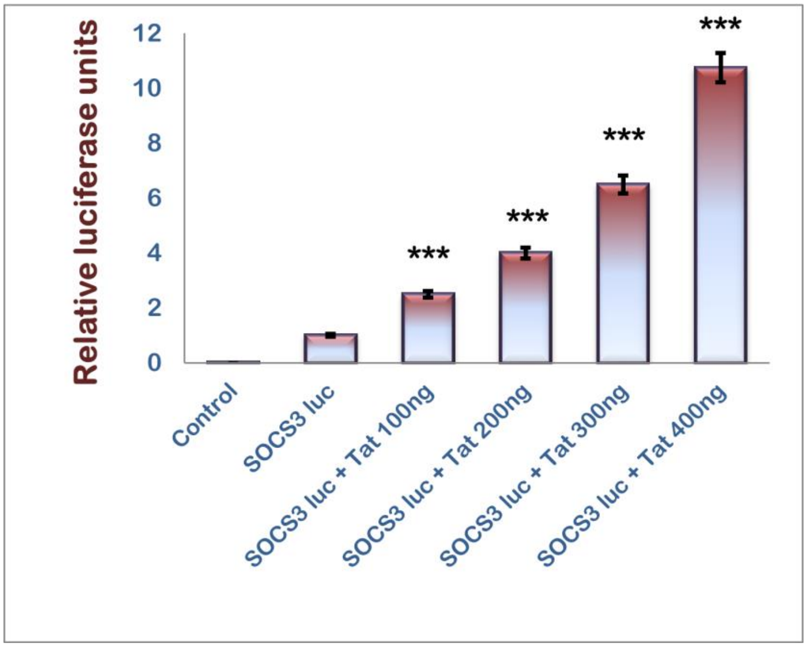

Supplementary Figure 3 HEK-293T cells were transfected with SOCS3-luc reporter plasmid (100ng), renilla luciferase along with different doses of Myc-Tat as shown. Empty vector was used to equalize the amount of DNA transfected. After 24 hrs of transfection, luciferase activity was assayed using dual luciferase assay reagents by luminometer. The values represent the mean +SEM of three independent experiments. P value was calculated by student T test (*p<0.05, **p<0.01, ***p<0.001)

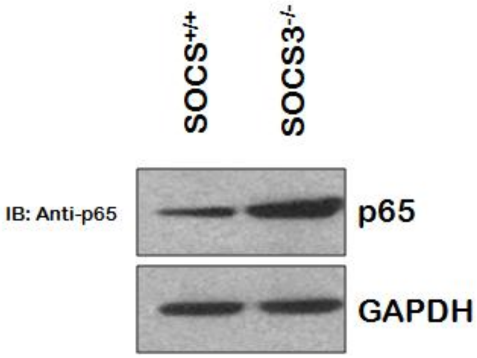

Supplementary Figure 4 SOCS3$^{+/+}$ and SOCS3$^{-/-}$ MEFs were lysed and cell lysates were analyzed for p65 using anti-p65 antibody. GAPDH was probed as loading control using anti-GAPDH antibody.

References:

1. Klave B, Berkhout B. Comparison of the 5′ and 3′ LTR promoter function in the human immunodeficiency virus. *J. Virol.* 1994;68,:3830–3840

2. Adachi A, Gendelman HE, Koenig S, et al. Production of acquired immunodeficiency syndrome-associated retrovirus in human and nonhuman cells transfected with an infectious molecular clone. *J. Virol.* 1986;59:284–291

3. Chang L-J, Urlacher V, Iwakuma T, Cui Y, Zucali J. Efficacy and safety analyses of a recombinant human immunodeficiency virus type 1 derived vector system. *Gene Ther.* 1999;6:715–728

4. Xirodimas DP, Stephen CW, Lane DP. Co-compartmentalization of p53 and Mdm-2 is a major determinant for Mdm-2-mediated degradation of p53. *Exp. Cell Res.* 2001;270:66–77

5. Childs KS, Andrejeva J, Randall RE, Goodbourn S. Mechanism of mda-5 Inhibition by Paramyxovirus V Proteins. *J. Virol.* 2009;83(3):1465–1473

6. Moreno R, Sobotzik J, Schultz C, Schmitz ML. Specification of the NF-κB transcriptional response by p65 phosphorylation and TNF-induced nuclear translocation of IKKε. *Nucleic Acids Res***.** 2010;38(18):6029–6044

7. Jiang Z, Georgel P, Li C, Choe J, et al. Details of Toll-like receptor:adapter interaction revealed by germ-line mutagenesis. *Proc. Natl. Acad. Sci. USA.* 2006;103(29):10961-10966

8. Ballard DW, Dixon EP, Peffer NJ, et al. The 65-kDa subunit of human NF-kappa B functions as a potent transcriptional activator and a target for v-Rel-mediated repression. *Proc. Natl. Acad. Sci. USA.* 1992;89(5):1875- 1879

9. Chen LF, MuY, Greene WC. Acetylation of RelA at discrete sites regulates distinct nuclear functions of NF-kappaB. *EMBO J.* 2002;21(23):6539-6548
